# Immunogenicity and safety of a multi-human dose formulation of Biological E’s 14-valent pneumococcal polysaccharide conjugate vaccine (PNEUBEVAX 14^®^) administered to 6–8-week-old healthy infants: a phase 3, single-blind, randomized, active-controlled study

**DOI:** 10.3389/fimmu.2025.1550227

**Published:** 2025-04-07

**Authors:** Subhash Thuluva, Ramesh V. Matur, Subbareddy Gunneri, Rammohan Reddy Mogulla, Kamal Thammireddy, Kalyan Kumar Peta, Piyush Paliwal, Niranjana S. Mahantshetti, Ramesh Kumar Banala, Prashanth Siddaiah

**Affiliations:** ^1^ Departments of R&D and Clinical Development, Biological E Limited, Hyderabad, Telangana, India; ^2^ Department of Paediatrics, KLES Dr. Prabhakar Kore Hospital & Medical Research Centre, Belgaum, Karnataka, India; ^3^ Department of Paediatrics, King George Hospital, Visakhapatnam, Andhra Pradesh, India; ^4^ Department of Paediatrics, Cheluvamba Hospital, Mysuru, Karnataka, India

**Keywords:** vaccine, pneumococcal conjugate vaccine, PCV, serotype 6A, infant immunization

## Abstract

**Background:**

Pneumococcal conjugate vaccines (PCVs) have considerably reduced the burden of invasive pneumococcal disease (PD) worldwide. Consequently, though, there has been an increase in non-vaccine serotype-induced PD particularly at both the extremes of age. Biological E has developed a 14-valent PCV (PNEUBEVAX 14®) that includes additional serotypes 22F and 33F. PNEUBEVAX 14® was shown to be safe, immunogenic, and non-inferior to Prevenar-13® (PCV-13) when administered to infants in a pivotal phase 3 trial. In this study, the multi-dose presentation of PNEUBEVAX 14® with 2-phenoxyethanol as a preservative was assessed for safety and immunogenicity in infants.

**Methods:**

This was a phase 3, single-blind, randomized, active-controlled study in 6–8-week-old healthy infants, conducted at three sites across India. The safety and immunogenicity of multi-dose presentation of PNEUBEVAX 14® were assessed in a 6–10–14-week dosing schedule, with 300 infants randomized to receive either PNEUBEVAX 14® or PCV-13. Safety-wise solicited local reactions and systemic events, unsolicited adverse events (AEs), serious AEs, and medically attended AEs (MAAEs) were recorded and analyzed. Immunogenicity was assessed by measuring anti-pneumococcal capsular polysaccharide (anti-PnCPS) immunoglobulin G (IgG) antibodies for all 14 serotypes, as well as cross-reactivity to serotype 6A.

**Findings:**

The safety aspects of the multi-dose presentation of PNEUBEVAX 14® and PCV-13 were comparable with 23.3% of subjects having AEs in each of the two arms. There were no serious AEs, medically attended AEs, or deaths in either of the two study arms. Reported AEs were mild and solicited in nature, with injection site swelling and injection site pain being the most common AEs in both arms. The multi-dose presentation of PNEUBEVAX 14® was found to induce a robust immune response, including the new serotypes 22F and 33F. Importantly, PNEUBEVAX 14® also induced cross-reactive antibodies against serotype 6A.

**Interpretation:**

The multi-dose presentation of PNEUBEVAX 14® is both safe and immunogenic when administered to 6–8-week-old infants in a 6–10–14-week dosing schedule. These results extend the findings of a pivotal phase 3 study of the single-dose presentation of PNEUBEVAX 14® that showed that it was safe, robustly immunogenic, and non-inferior to PCV-13 in the same age group and dosing schedule. Taken together, these data suggest that both the single-dose and multi-dose presentations of PNEUBEVAX 14® can be safely administered to infants to prevent pneumococcal disease caused by *Streptococcus pneumoniae*.

**Clinical Trial Registration:**

https://ctri.nic.in/Clinicaltrials/pmaindet2.php?EncHid=NTk0MzA=&Enc=&userName=, identifier CTRI/2021/10/037067.

## Introduction

*Streptococcus pneumoniae*, a gram-positive, encapsulated bacterium, poses a significant global health threat, particularly affecting children below 5 years, adults over 50 years, and immunocompromised individuals. Also known as Pneumococcus, it remains a leading cause of non-invasive and invasive diseases, including acute otitis media (AOM), sinusitis, bacteremia, meningitis, and pneumonia (1). It is estimated that approximately 1 million children die of pneumococcal disease every year, mainly in African and Asian countries (2). Pneumonia and Invasive pneumococcal disease (IPD) such as meningitis and bacteremia is particularly devastating for children below the age of 1 year (http://www.cdc.gov/abcs/reports-finds/surv-reports.html).

Worldwide, approximately 90 distinct pneumococcal serotypes have been described, characterized by the composition of their polysaccharide capsules. Despite the existence of over 90 serotypes of *S. pneumoniae*, only a few are responsible for causing disease, and these can vary with geography and age (3). Alarmingly, more than 30% of cases that involve pneumococci are resistant to one or more antibiotics (4).

Vaccines that target multiple pneumococcal serotypes have been effective in preventing pneumococcal disease. The 23-valent polysaccharide vaccine (PPSV23; Pneumovax^®^ 23, Merck and Co., Inc., NJ, USA) is the most widely used vaccine globally in adults, offering protection against 23 different pneumococcal serotypes. However, it has poor immunogenicity in those aged <2 years, as it induces T cell-independent immune responses (5). The introduction of pneumococcal polysaccharide–protein conjugate vaccines (PCVs) such as PCV-7 (Prevenar 7^®^), PCV-10 (PNEUMOSIL^®^ and SYNFLORIX^®^), and PCV-13 (Prevenar 13^®^) has significantly reduced the worldwide incidence of IPD across all age groups (6). Mechanistically, PCVs have been shown to trigger T cell-dependent immune responses against specific serotypes, providing robust and long-term protection against IPD, particularly in younger children (6–9).

Infant immunization programs with PCVs have significantly reduced the overall incidence of vaccine-type IPD cases worldwide (>90% reduction) (6, 10). However, disease-causing non-vaccine *S. pneumoniae* serotypes continue to replace the vaccine types in response to the introduced PCVs. Hence, the need for ongoing development of new PCVs with extended coverage against recently emerging and clinically relevant serotypes remains crucial to enhance global protection.

Biological E Limited has developed a PCV, PNEUBEVAX 14®, covering 14 different serotypes, including serotypes 22F and 33F, which are not present in PCV-10 or PCV-13. Increasing accessibility to PCVs with additional serotypes, especially in countries like India with a high disease burden, holds promise in reducing the impact of IPD. PNEUBEVAX 14®, in its single human dose presentation, has been extensively studied in phase 1, phase 2, and phase 3 clinical trials; its safety and immunogenicity have been established (CTRI/2017/06/008759, CTRI/2017/11/010387, and CTRI/2020/02/023129). Apart from the single-dose formulation, there is a need to establish the safety and immunogenicity of a multi-dose formulation of BE-PCV-14. Such multi-dose formulations are cost-effective, increase logistical effectiveness, and facilitate large immunization campaigns. Here, we report findings from a phase 3 study evaluating the safety and immunogenicity of a multi-human dose presentation of PNEUBEVAX 14® administered to 6–8-week-old healthy infants (N = 300) in a 6–10–14-week dosing schedule.

## Methods

### Study design and participants

This phase-3, two arm, single blind, randomized, active-controlled study was conducted between November 2021 and March 2022 in India, across six sites: KLES Dr. Prabhakar Kore Hospital & Medical Research Centre, Belgaum; King George Hospital, Visakhapatnam; and Cheluvamba Hospital, Mysore. It was designed to demonstrate the safety and immunogenicity of multi-human dose formulation of Biological E’s 14-valent pneumococcal polysaccharide conjugate vaccine containing 2-phenoxyethanol as a preservative, compared with that of Prevenar-13™ (PCV-13) in 6–8-week-old healthy infants of either gender.

Participants were equally randomized (1:1) to receive either the multi-dose presentation of PNEUBEVAX 14® (BE-PCV-14) or PCV-13. In total, 300 subjects were enrolled in the study (n = 150 in the BE-PCV-14 arm and n = 150 in the PCV-13 arm). A dose of 0.5 mL of the study vaccine (BE-PCV-14 or PCV-13) was administered intramuscularly in a three-dose vaccination schedule (3 + 0) with a 4-week interval between doses.

All enrolled subjects (N = 300) were healthy infants ≥3,300 g of weight at the time of screening. None of the participants were vaccinated with any licensed or investigational pneumococcal vaccine prior to enrollment. Vaccinations [Bacillus Calmette–Guérin (BCG), hepatitis B, diphtheria, tetanus, whole-cell pertussis, *Haemophilus influenzae* type b (DTwP-HepB-Hib), polio, and rotavirus vaccines] under the expanded program on immunization (EPI) schedule were allowed to be administered as per the age of the participant(s). Informed consent was obtained from the subjects’ parent(s)/legally authorized representative (LAR) prior to performing any study-specific procedure. Exclusion criteria used were evidence of prior *S. pneumoniae* infection, use of any investigational or non-registered product up to 30 days prior to study vaccine administration, history of any neurological disorders, family history of congenital or hereditary immunodeficiency, and history of allergic disease or known hypersensitivity likely to be exacerbated by any component of the study vaccines. A complete list of eligibility criteria is provided as supplementary information.

The study was conducted in accordance with the Declaration of Helsinki, the International Conference on Harmonization guidelines (Good Clinical Practices), and the new drug and clinical trial rules, 2019 (11). The protocol was approved by the institutional review board or ethics committee at each study site.

#### Randomization and masking

Eligible subjects were randomized to receive either BE-PCV-14 or PCV-13 in a 1:1 ratio. Randomization numbers were assigned using an Interactive Web Response System (IWRS) with sequence generated using the SAS software (SAS Institute Inc., Cary, NC, USA).

### Procedure

Screening, enrolment, and first dose of primary vaccination were performed when infants were 6–8 weeks old (visit 1, day 0). The second and third doses of primary vaccination (visit 2, day 28; and visit 3, day 56) and a final follow-up immunogenicity visit (visit 4, day 86) took place at 4 weeks’ intervals.

For the second, third, and fourth visits, an additional time window of +7 days was permitted to ensure the subject’s visit compliance.

A multi-human dose preparation of 2.5 mL supporting the administration of five doses of vaccine was used in this study. Each 0.5-mL single dose of BE-PCV-14 (PNEUBEVAX 14®) contained 3 μg of serotype 1; 2.2 μg of each of serotypes 3, 4, 5, 7F, 9V, 14, 18C, 19A, 19F, 22F, 23F, and 33F; and 4.4 μg of serotype 6B polysaccharides conjugated to approximately 35 μg of non-toxic diphtheria toxin cross-reacting material 197 (CRM_197_) protein and adsorbed onto ≤0.75 mg of aluminum phosphate (Al+++) and 4 mg of 2-phenoxyethanol as a preservative. Pfizer’s Prevenar-13™ 13-valent pneumococcal polysaccharide conjugate vaccine “PCV-13” (control vaccine) contained 2.2 μg of each of serotypes 1, 3, 4, 5, 6A, 7F, 9V, 14, 18C, 19A, 19F, and 23F saccharides; 4.4 μg of 6B saccharide; 34 μg CRM_197_ carrier protein; and 125 μg aluminum as aluminum phosphate adjuvant. Both vaccines were administered intramuscularly in the anterolateral aspect of the thigh.

Approximately 5.0 mL of blood was collected at visit 1 (day 0) and again at visit 4 (day 86). Anti-pneumococcal capsular polysaccharide (anti-PnCPS) immunoglobulin G (IgG) antibody concentration estimation against each of the 14 vaccine serotypes was performed as per the World Health Organization (WHO) reference ELISA (12) with a minor modification. Instead of cell wall polysaccharide (CWPS), CWPS-multi was used in the assay. Also, 22F pneumococcal polysaccharide (PnPS) was used for pre-adsorption to neutralize non-specific antibodies and other common contaminants present in the PnPS coating antigens.

#### Outcomes

The primary outcome of the study was to demonstrate the safety of BE-PCV-14 in comparison with PCV-13. This outcome was measured by the incidence rates of local and systemic reactogenicity events, adverse events (AEs), medically attended AEs, and serious adverse events (SAEs).

The secondary objectives included assessing the immune response induced by 12 common serotypes (1, 3, 4, 5, 6B, 7F, 9V, 14, 18C, 19A, 19F, and 23F) of BE-PCV-14 and PCV-13, as measured by vaccine serotype-specific IgG concentrations 1 month after the completion of primary series of vaccination. Additionally, the immune response induced by the two new serotypes 22F and 33F in BE-PCV-14, which are not present in PCV-13, were assessed in comparison with any serotype in the PCV-13 that achieved the lowest percentage of participants with IgG ≥ 0.35 μg/mL at 1 month after the third dose. Additionally, cross-reactive immune response to serotype 6A were also assessed by measuring serotype-specific IgG concentration.

### Sample size determination

The phase 3 comparative study was primarily designed as a descriptive comparison of the safety and immunogenicity of BE-PCV-14 in 6–8-week-old healthy infants in a 3 + 0 dosing schedule in comparison with Pfizer’s PCV-13 vaccine. Although there was a comparator group, this descriptive study was not formally powered to detect differences in treatment effects between investigational and comparator vaccines. The treatment effect has already been evaluated in a pivotal phase 3 clinical study where the single-dose formulation of BE-PCV-14 was shown to be both safe and immunogenic (13). The main focus was, therefore, to make “as good a decision as possible” with a medium sample size of 300 subjects randomized equally between the two groups but leave the “proof of using multi human dose presentation”. Accordingly, a sample size of 150 subjects in each treatment arm was considered adequate, and the total sample to be enrolled was 300 healthy infants.

### Statistical analyses

All demographic and baseline characteristics of both groups were presented using an intention-to-treat analysis population. The intention-to-treat (ITT) analysis population was defined to include all subjects randomized into the study. All the safety analyses were desccriptive. Demographics, and baseline characteristics were analyzed by summary statistics. For continuous variables, n, mean, standard deviation, median, and range (minimum and maximum) were presented. For categorical data, frequencies were computed. All reported adverse events during the entire study period were summarized by calculating frequencies and were listed per subject including severity and relationship to the vaccine (causality).

The number and percentage of subjects with AEs were presented by system organ class (SOC) and preferred term (PT). All medically attended AEs (MAAEs) reported during the study were listed and analyzed for expectedness and causality. Two-sided 95% exact confidence intervals (CIs) were calculated for all the occurrence rates of reported AEs and SAEs during the study. All AEs were coded using the Medical Dictionary for Regulatory Activities (MedDRA™; version 25.1) coding dictionary, and concomitant medications were coded using the WHO Drug Dictionary, March 2022.

The secondary objective of the study was to demonstrate the serotype-specific immunogenicity of BE-PCV-14 in comparison with PCV-13. Immunogenicity assessment was based on the per-protocol population (PP). Serum anti-PnCPS IgG antibody concentrations were measured in μg/mL against each of the vaccine serotypes in both vaccine groups and were estimated by a validated IgG ELISA method.

The proportion of subjects with serotype-specific anti-PnCPS IgG antibody concentration ≥0.35 μg/mL (seroconversion threshold) was calculated in both groups. Geometric mean concentration (GMC) of IgG antibodies against each of the vaccine serotypes along with their two-sided 95% CIs were calculated pre-vaccination and again 1 month after the third dose vaccination. For all GMC calculations, natural log transformation was used, and the results were back-transformed. The proportion of subjects achieving ≥2-fold and ≥4-fold rise in anti-PnCPS IgG antibody concentrations from the baseline against each of the vaccine serotypes was also calculated. The fold rise was calculated as the ratio of the post-vaccination concentration value to the pre-vaccination value against each of the vaccine serotypes along with their two-sided 95% CIs. Reverse cumulative distribution (RCD) curves of anti-PnCPS antibody concentrations by serotype were plotted for all common vaccine serotypes in both groups. Bar graphs with 95% confidence intervals were included to represent data wherever relevant.

## Results

**Figure 1:**
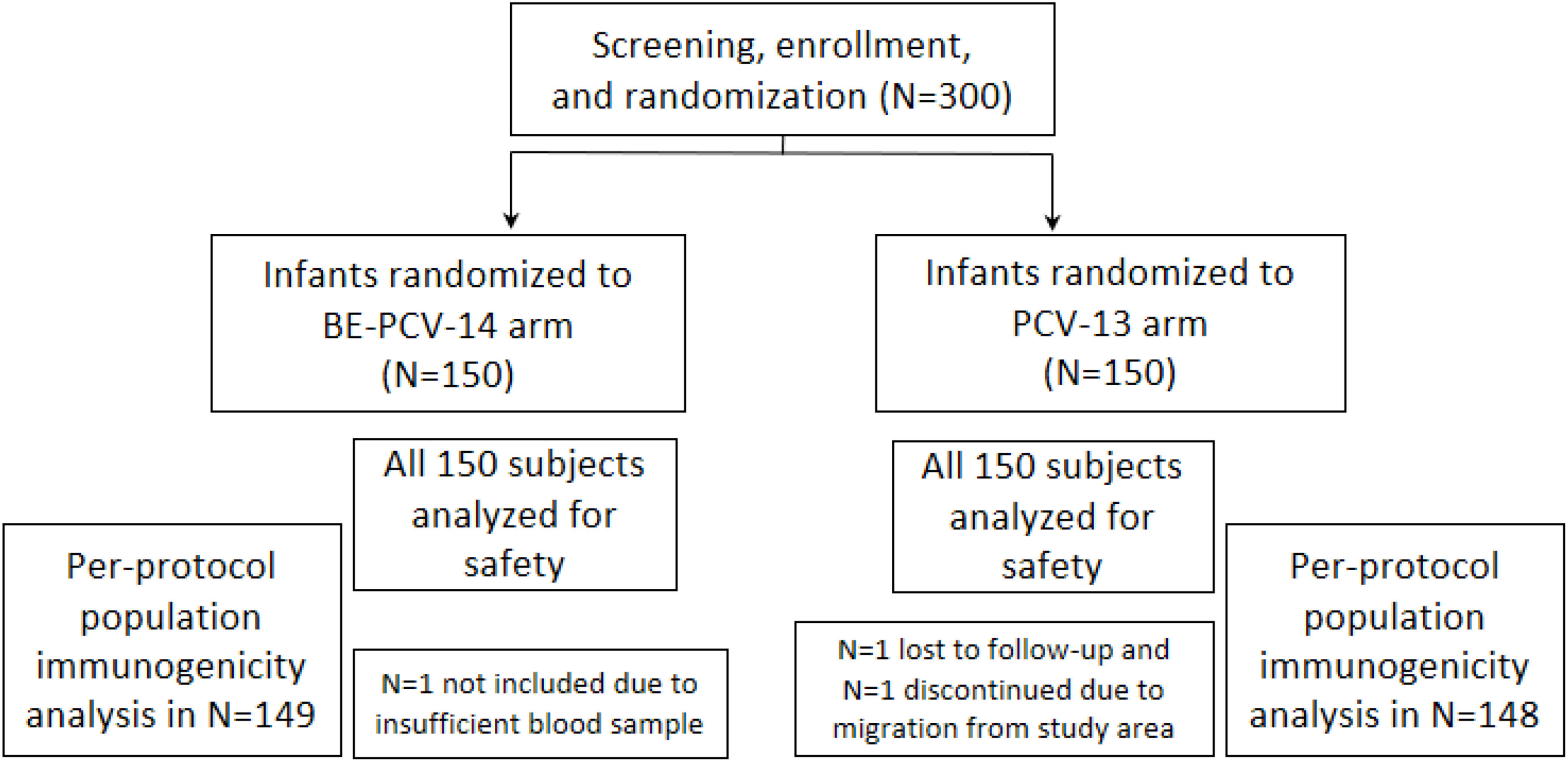
Subject disposition. n, number; PCV, pneumococcal conjugate vaccine; BE-PCV-14: Biological E’s 14-valent PCV; PCV-13: Pfizer’s 13-valent PCV.

A total of 300 subjects were screened and randomly assigned to receive either BE-PCV-14 (n = 150) or PCV-13 (n = 150) study vaccines. Safety analyses were performed in all enrolled subjects (n = 300), whereas immunogenicity (per-protocol population) was completed in 297 subjects. Out of 300 subjects, 297 (99.0%) subjects (149 in the BE-PCV-14 arm and 148 in the PCV-13 arm) completed all the visit-specific procedures and were therefore included in the immunogenicity analysis. Two subjects dropped out from the study, while one study participant was excluded from the per-protocol population due to insufficient serum sample collection. Subject disposition with the reason for dropout from the study is presented in **Figure 1**. The demographics of the intention-to-treat population are presented in **Table 1**. The age of the study subjects was comparable in both the BE-PCV-14 and PCV-13 arms. The summary of other eligible vaccination status and concomitant medications received by study subjects are presented in **Supplementary Tables 1**, **2**, respectively.

**Table 1:** Demographic characteristics of study participants.

Parameter/statistics/category	BE-PCV-14 (N = 150)	PCV-13 (N = 150)	Overall (N = 300)
Age (days)			
N1	150	150	300
Mean	48.2	48.3	48.3
SD	3.06	3.24	3.15
Median	48.0	48.0	48.0
Q1:Q3	(46.0:50.0)	(46.0:51.0)	(46.0:50.0)
Range (min:max)	(42.0:56.0)	(42.0:56.0)	(42.0:56.0)
Gender, N1 (%)			
Male	80 (53.3%)	99 (66.0%)	179 (59.7%)
Female	70 (46.7%)	51 (34.0%)	121 (40.3%)
Length (cm)			
N1	150	150	300
Mean	53.4	53.5	53.5
SD	3.20	3.08	3.14
Median	54.0	54.0	54.0
Q1:Q3	(50.0:56.0)	(50.1:56.0)	(50.0:56.0)
Range (min:max)	(46.0:63.0)	(48.0:60.0)	(46.0:63.0)
Weight (h)			
N1	150	150	300
Mean	4,164.6	4,189.3	4,177.0
SD	459.79	400.70	430.71
Median	4,050.0	4,100.0	4,100.0
Q1:Q3	(3,900.0:4,400.0)	(3,900.0:4,400.0)	(3,900.0:4,400.0)
Range (min:max)	(3,300.0:5,500.0)	(3,300.0:5,300.0)	(3,300.0:5,500.0)
Nationality, N1 (%)			
Indian	150 (100.0%)	150 (100.0%)	300 (100.0%)
BMI			
N1	150	150	300
Mean	14.7	14.7	14.7
SD	1.61	1.71	1.66
Median	14.8	14.8	14.8
Q1:Q3	(13.6:15.8)	(13.4:15.9)	(13.4:15.9)
Range (min:max)	(10.7:20.8)	(11.0:19.6)	(10.7:20.8)

Percentages were calculated using respective column header count as denominator.

N1, subject count; N, sample size.

### Safety findings

**Figure 2:**
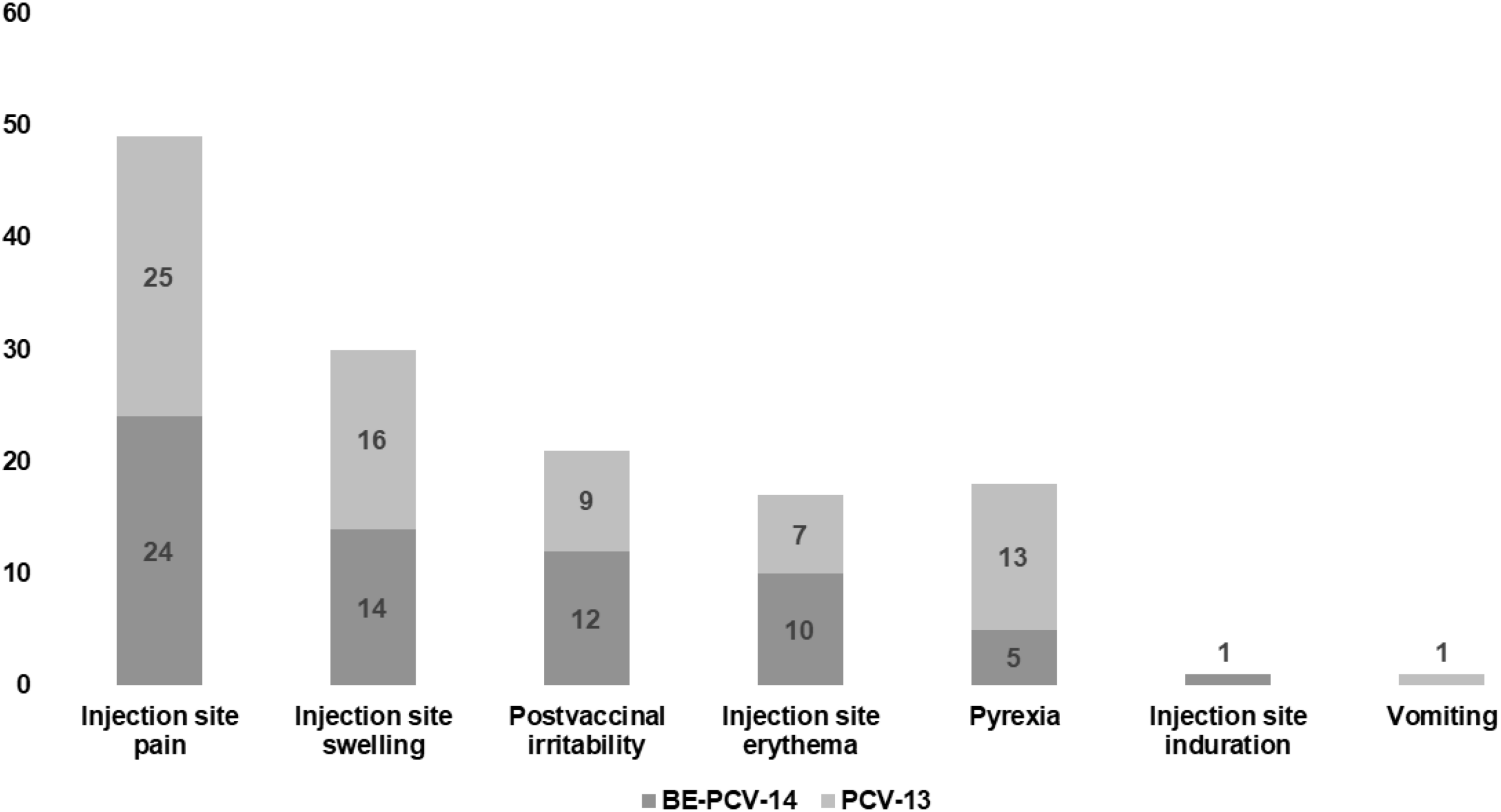
Comparison of AEs in the two test groups, with the Y-axis representing the number of AEs. AEs, adverse events.

Among all vaccinated infants (n = 300), 23.3% of subjects in each of the two arms reported at least one adverse event. All of these reported adverse events were solicited in nature and related to the study vaccines. The most common AEs reported in both groups were injection site pain (11.3% subjects in the BE-PCV-14 arm and 12.7% subjects in the PCV-13 arm) and swelling (6.7% subjects in the BE-PCV-14 arm and 8.7% subjects in the PCV-13 arm) (**Figure 2**). All reported AEs were mild in intensity. There were no reports of severe, serious, or medically attended adverse events in either of the vaccine groups. An overview of all reported solicited AEs is presented in **Table 2**, and a summary of AEs by SOC and PT is presented in **Table 3**. An additional overview of AEs by severity grade and causality is presented in **Table 4**. A summary of local, solicited systemic, and unsolicited adverse events by SOC and PT are provided in **Supplementary Tables 3**-**5**. The vital signs of study subjects did not change over time, and the results of physical examinations did not reveal any safety concerns of significance. Overall, the safety profile of BE-PCV-14 was comparable to that of the licensed control vaccine PCV-13.

**Table 2:** Overview of AEs—safety population (N = 300).

Category N1 (%) [95% CI] n	BE-PCV-14 (N = 150)	PCV-13 (N = 150)
Overall AEs	35 (23.3%) [16.82:30.93] 66	35 (23.3%) [16.82:30.93] 71
Serious AEs	0 (0.0%) [NE] 0	0 (0.0%) [NE] 0
Severe AEs	0 (0.0%) [NE] 0	0 (0.0%) [NE] 0
Local AEs	30 (20.0%) [13.92:27.30] 49	30 (20.0%) [13.92:27.30] 48
Systemic AEs	10 (6.7%) [3.24:11.92] 17	14 (9.3%) [5.20:15.16] 23
Medically Attended AEs	0 (0.0%) [NE] 0	0 (0.0%) [NE] 0
AEs within 60 minutes post-vac	2 (1.3%) [0.16:4.73] 3	0 (0.0%) [NE] 0
AEs within 7 days post-vac	35 (23.3%) [16.82:30.93] 66	35 (23.3%) [16.82:30.93] 71
AEs between day 0 and day 28	23 (15.3%) [9.98:22.11] 41	18 (12.0%) [7.27:18.30] 32
AEs between day 28 and day 56	11 (7.3%) [3.72:12.74] 18	16 (10.7%) [6.22:16.74] 20
AEs between day 56 and day 86	7 (4.7%) [1.90:9.38] 7	15 (10.0%) [5.71:15.96] 19
Any solicited AEs	35 (23.3%) [16.82:30.93] 66	35 (23.3%) [16.82:30.93] 71
Any unsolicited AEs	0 (0.0%) [NE] 0	0 (0.0%) [NE] 0

Percentages were calculated using respective column header group count as denominator. 95% CI was calculated by Clopper–Pearson method.

N1, subject count; N, subject count; N, sample size; n, event count; AEs, adverse events.

General note: All AEs were presented as follows: Subject count (Percentage of subjects) [95% CI] Event count. Solicited local and systemic AEs were recorded during 7 days (days 0–6) after the vaccination. Unsolicited adverse events were reported at any time until 28 days after the vaccination.

**Table 3:** Summary of AEs by SOC and PT—safety population (N = 300).

SOC/PT, N1 (%) [95% CI] n	BE-PCV-14 (N = 150)	PCV-13 (N = 150)
Overall	35 (23.3%) [16.82:30.93] 66	35 (23.3%) [16.82:30.93] 71
Gastrointestinal disorders	0 (0.0%) [NE] 0	1 (0.7%) [0.02:3.66] 1
Vomiting (mild)	0 (0.0%) [NE] 0	1 (0.7%) [0.02:3.66] 1
General disorders and administration site conditions	35 (23.3%) [16.82:30.93] 66	35 (23.3%) [16.82:30.93] 70
Injection site erythema	10 (6.7%) [3.24:11.92] 10	7 (4.7%) [1.90:9.38] 7
Injection site induration	1 (0.7%) [0.02:3.66] 1	0 (0.0%) [NE] 0
Injection site pain	17 (11.3%) [6.74:17.52] 24	19 (12.7%) [7.80:19.07] 25
Injection site swelling	10 (6.7%) [3.24:11.92] 14	13 (8.7%) [4.70:14.36] 16
Irritability post-vaccination	5 (3.3%) [1.09:7.61] 12	6 (4.0%) [1.48:8.50] 9
Pyrexia	5 (3.3%) [1.09:7.61] 5	12 (8.0%) [4.20:13.56] 13

Percentages were calculated using column header group count as denominator. 95% CIs were calculated by Clopper–Pearson method.

N1, subject count; N, sample size; n, event count; AEs, adverse events; SOC, system organ class; PT, preferred term.

General Note: All AEs were presented as follows: Subject count (Percentage of subjects) [95% CI] Event count.

**Table 4:** Overview AEs by severity and causality—safety population (N = 300).

Category N1 (%) [95% CI] n	BE-PCV-14 (N = 150)	PCV-13 (N = 150)
Number of subjects with at least one AE	35 (23.3%) [16.82:30.93] 66	35 (23.3%) [16.82:30.93] 71
Number of subjects with at least one SAE	0 (0.0%) [NE] 0	0 (0.0%) [NE] 0
Number of subjects with at least one MAAE	0 (0.0%) [NE] 0	0 (0.0%) [NE] 0
Severity		
Mild	35 (23.3%) [16.82:30.93] 66	35 (23.3%) [16.82:30.93] 71
Moderate	0 (0.0%) [NE] 0	0 (0.0%) [NE] 0
Severe	0 (0.0%) [NE] 0	0 (0.0%) [NE] 0
Life-threatening	0 (0.0%) [NE] 0	0 (0.0%) [NE] 0
Causality		
Related (certain/probable/possible)	35 (23.3%) [16.82:30.93] 66	35 (23.3%) [16.82:30.93] 71
Unrelated (unlikely/unrelated/unclassifiable)	0 (0.0%) [NE] 0	0 (0.0%) [NE] 0
Number of subjects discontinued due to AE	0 (0.0%) [NE] 0	0 (0.0%) [NE] 0

AE, adverse event; SAE, serious AE; MAAE, medically attended AE.

### Immunogenicity findings

**Figure 3:**
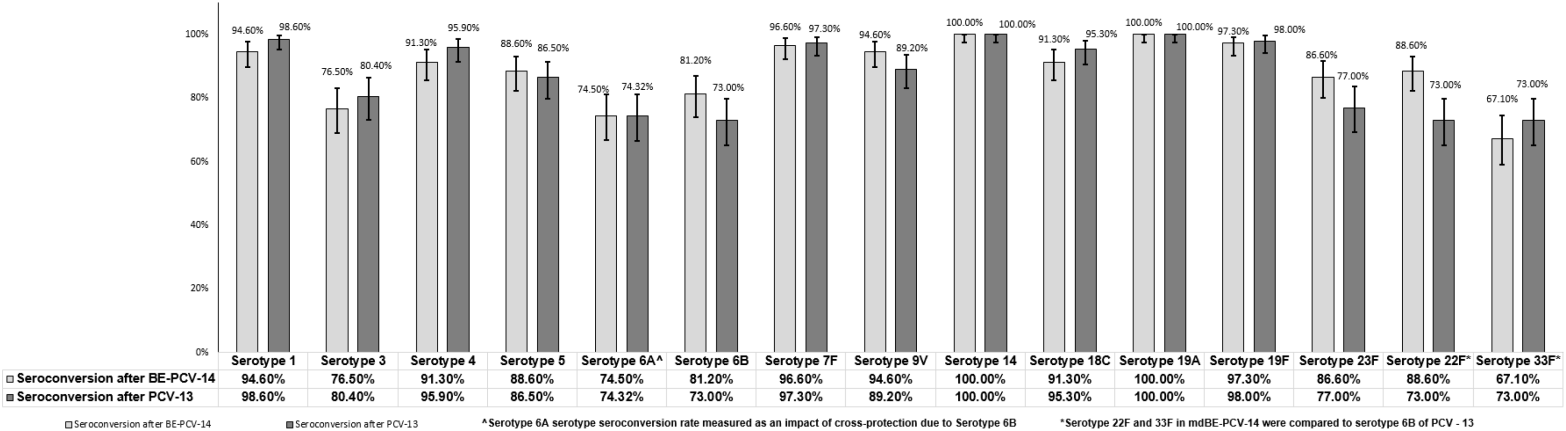
Difference in proportion of seroconversion rates with 95% confidence interval. CI, confidence interval; IgG, immunoglobulin G; GMC, geometric mean concentration.

Immunogenicity assessments were conducted as secondary objectives of this study. At day 86 (1 month after the third dose), the proportion of subjects in the BE-PCV-14 group (n = 149) with anti-PnCPS IgG antibody concentrations at or above the protective threshold (≥0.35 µg/mL) ranged from 76.5% (serotype 3) to 100% (serotypes 14 and 19A). In the PCV-13 group (n = 148), the response rate varied from 73% (serotype 6B) to 100% (serotypes 14 and 19A). Seroconversion rates were comparable in the two study arms and are presented in **Figure 3**. Seroconversion rates for serotypes 22F and 33F that are present only in the BE-PCV-14 group were compared with those for the lowest-performing serotype 6B in the PCV-13 group (**Figure 3**).

**Figure 4:**
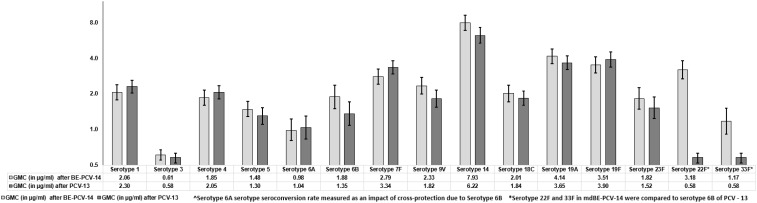
Geometric mean concentrations with 95% confidence intervals. CI, confidence interval; IgG, immunoglobulin G; GMC, geometric mean concentration.

**Figure 5:**
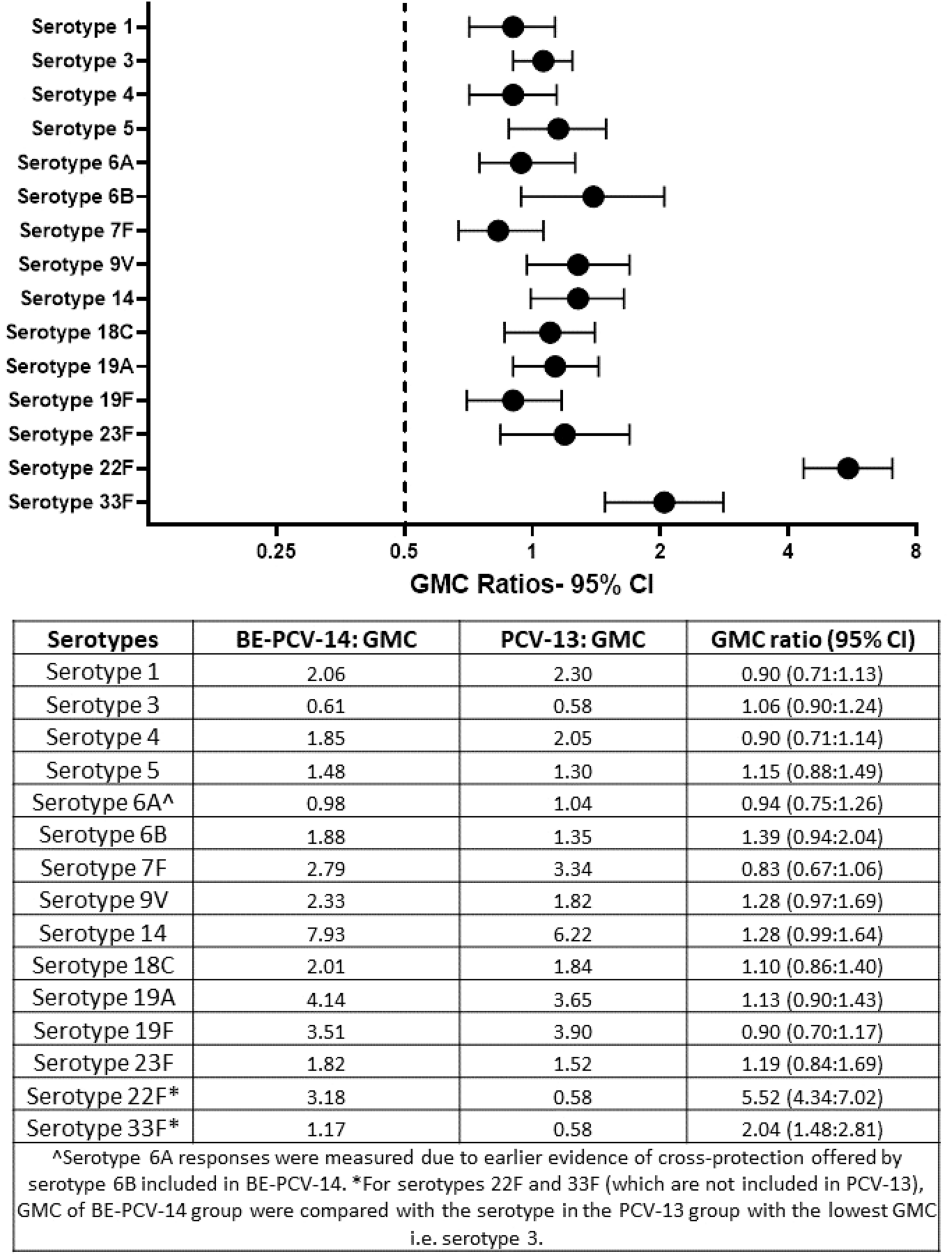
Geometric mean concentration ratios of test to control with 95% confidence intervals. CI, confidence interval; IgG, immunoglobulin G; GMC, geometric mean concentrations.

The GMCs of anti-PnCPS IgG were estimated and found to be comparable in BE-PCV-14 or PCV-13. GMC were in the range of 0.61 (serotype 3) to 7.93 (serotype 14) and 0.58 (serotype 3) to 6.22 (serotype 14) for the 12 common serotypes present in the BE-PCV-14 and PCV-13 treatment groups, respectively (**Figure 4**). The GMCs of serotypes 22F and 33F were compared to those of the lowest-performing serotype in PCV-13 (serotype 3). As an additional *post hoc* analysis, the GMC ratios for BE-PCV-14 to PCV-13 were calculated, and the lower limit of the 95% CIs for all of the serotypes was found to be above 0.5 (**Figure 5**). This is in accordance with WHO Technical Report Series 977 guidelines (14), meeting the non-inferiority criteria with those serotypes in PCV-13 in terms of GMC ratios (≥0.5).

**Figure 6:**
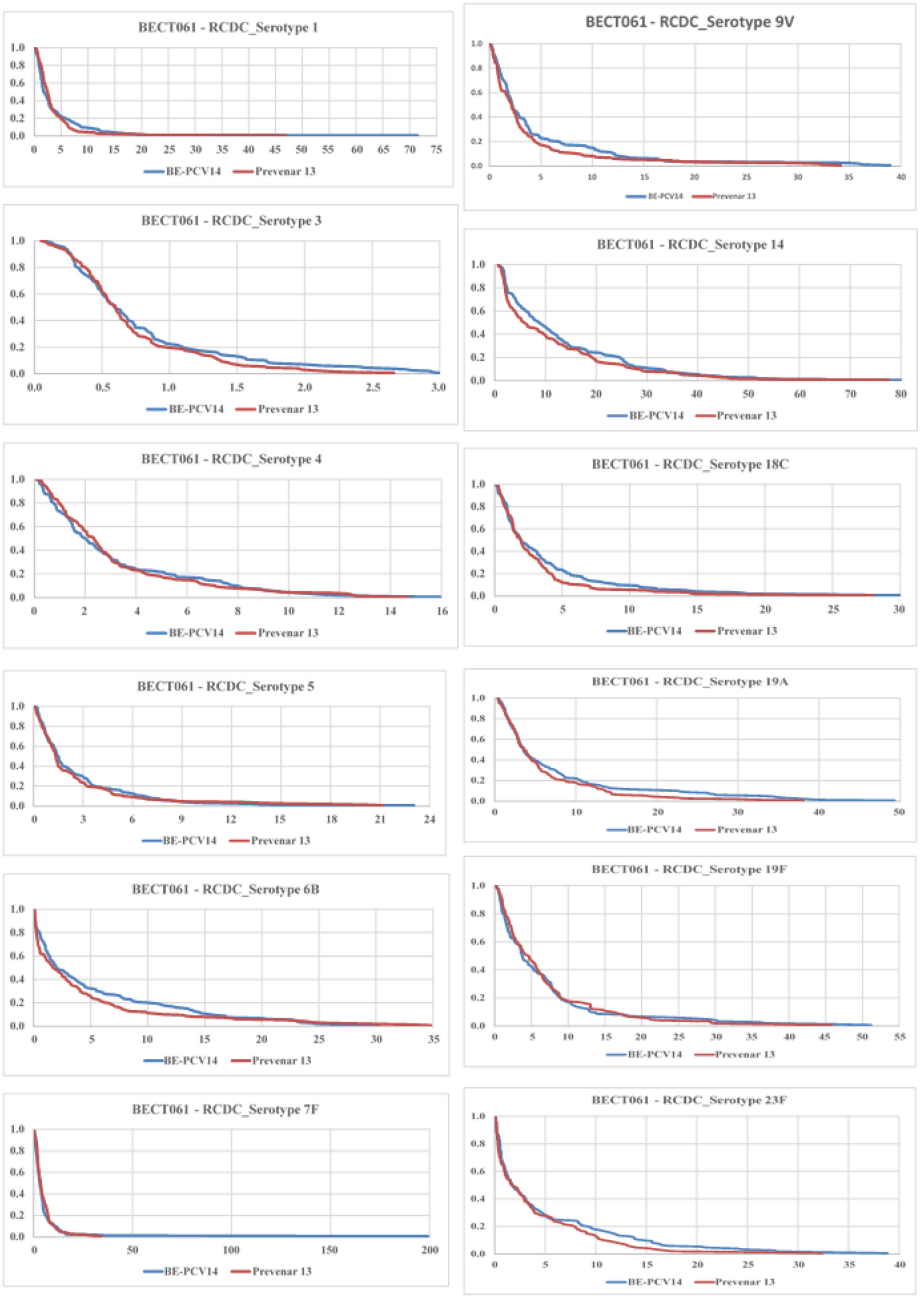
Reverse cumulative distribution (RCD) curves of anti-PnCPS IgG antibody concentrations for 12 common serotypes between BE-PCV-14 and PCV-13. RCD, reverse cumulative distribution; PnCPS IgG, pneumococcal conjugate polysaccharide-specific immunoglobulin G; GMC, geometric mean concentration.

The proportion of subjects achieving ≥4-fold increase in anti-PnCPS IgG antibody concentrations at day 86 from baseline is presented in **Table 5**. The reverse cumulative distribution curves of all serotype IgGs showed nearly identical curves post-vaccination with BE-PCV-14 or PCV-13 (**Figure 6**).

**Table 5:** Proportion of subjects achieving ≥4-fold rise in anti-PnCPS IgG antibody concentrations—PP population (N = 297).

Anti-PnCPS IgG against each of the vaccine serotype post-vaccination (day 86)	BE-PCV-14 (N = 149) N1 (%) [95% CI]	PCV-13 (N = 148) N1 (%) [95% CI]
Serotype 1	102 (68.5%) [60.35:75.82]	111 (75.0%) [67.22:81.75]
Serotype 3	43 (28.9%) [21.74:36.84]	62 (41.9%) [33.84:50.27]
Serotype 4	69 (46.3%) [38.11:54.65]	86 (58.1%) [49.73:66.16]
Serotype 5	87 (58.4%) [50.04:66.40]	83 (56.1%) [47.69:64.22]
Serotype 6B	80 (53.7%) [45.35:61.89]	78 (52.7%) [44.34:60.96]
Serotype 7F	97 (65.1%) [56.87:72.72]	116 (78.4%) [70.87:84.72]
Serotype 9V	80 (53.7%) [45.35:61.89]	84 (56.8%) [48.37:64.87]
Serotype 14	23 (15.4%) [10.04:22.26]	27 (18.2%) [12.38:25.42]
Serotype 18C	39 (26.2%) [19.32:34.00]	40 (27.0%) [20.06:34.94]
Serotype 19A	33 (22.1%) [15.76:29.67]	44 (29.7%) [22.50:37.79]
Serotype 19F	71 (47.7%) [39.41:55.98]	75 (50.7%) [42.34:58.98]
Serotype 23F	69 (46.3%) [38.11:54.65]	76 (51.4%) [43.01:59.64]

Percentage was calculated using respective header count as denominator. 95% CI was calculated using Clopper–Pearson method.

PnCPS, pneumococcal capsular polysaccharide.

In this study, we tested the cross-protection against serotype 6A offered by serotype 6B for participants who received BE-PCV-14 (PNEUBEVAX 14®). Cross-protection was assessed by a ≥2-fold increase in the 6A-specific IgG antibody concentrations above the pre-vaccination levels. There was a 2.04-fold (pre-vaccination GMC, 0.48; post-vaccination GMC, 0.98) increase in serotype 6A-specific IgG concentration at day 86 from baseline (**Table 6**). Additionally, the geometric mean concentration ratio of IgG concentrations of the BE-PCV-14 arm to the PCV-13 arm met the *post hoc* non-inferiority criteria with a value of 0.94 and 95% confidence intervals from 0.75 to 1.26 (lower limit greater than 0.5). The seroconversion rate for serotype 6A after BE-PCV-14 was 74.50% and after PCV-13 was 74.32%, with the lower limit of the 95% confidence interval being −9.75%, which also met the *post hoc* non-inferiority criteria of being above −10% (**Table 6**).

**Table 6:** Fold rise in geometric mean concentration and seroconversion rate above pre-vaccination levels in serotype 6A—PP population (N = 297).

	GMC of anti-PnCPS IgG antibodies			Seroconversion rate		
	Pre-vaccination (day 0)	Post-vaccination (day 86)	Geometric mean concentration ratio	Pre-vaccination (day 0)	Post-vaccination (day 86)	Difference in proportions
BE-PCV-14	0.48	0.98	–	57.05%	74.50%	**-**
PCV-13	0.32	1.04	**0.94** **(0.75, 1.26)**	52.03%	74.32%	**0.18%** **(−9.75%, 10.10%)**

N, sample size; GMC, geometric mean concentration; PnCPS, pneumococcal capsular polysaccharide. Bold values indicate values met the *post hoc* non-inferiority criteria.

The current study demonstrated that multi-human dose presentation of BE-PCV-14 was found to have comparable safety and immunogenicity with PCV-13 when administered to infants in a three-dose schedule.

## Discussion

In this phase 3 trial, we established that the multi-dose presentation of BE-PCV-14 (md-BE-PCV-14) is both safe and immunogenic when administered to infants in a three-dose schedule. The safety profile of BE-PCV-14 was comparable to that of the PCV-13 in terms of overall AE rates, relatedness, and reported medically attended AEs. The proportion of subjects who experienced at least one adverse event was similar between the BE-PCV-14 and PCV-13 groups, both at approximately 23%.

The most commonly reported adverse events in the BE-PCV-14 group were injection site pain, swelling, and erythema. Other AEs reported included post-vaccination irritability, pyrexia, and injection site induration. All of these AEs also occurred in the PCV-13 arm with an additional case of vomiting. All reported AEs were mild in intensity, solicited in nature, and related to the study vaccine. No severe AEs or deaths were reported in either of the treatment groups. The BE-PCV-14 vaccine in multi-human dose presentation was well tolerated and found safe to be administered to infants.

These safety findings are in line with those of another phase 3 study conducted by Biological E, as well as other pneumococcal conjugate vaccines. In particular, our recent phase 3 pivotal study of 1,290 healthy infants (10.1101/2023.12.21.23300357) with an equal number of infants receiving BE-PCV-14 or PCV-13 showed a very similar safety profile, with injection site pain most frequently reported as a local adverse reaction and pyrexia as a systemic adverse reaction. Multiple clinical trials of BE-PCV-14 in over 3,000 individuals have had only two reported SAEs, both of which were adjudicated to be unrelated to the vaccine. Similar to BE-PCV-14, other PCVs have been extensively studied in clinical trial settings as well as in post-marketing settings and have been found not to have any major safety issues (15, 16). Overall, the safety profile of BE-PCV-14 appears to be very similar to that of PCV-7 and PCV-13, a vaccine for which there is approximately two decades of clinical experience (17), assuring a similar continued long-term safety for BE-PCV-14 as well. The multi-human dose preparation on trial in this study contained 2-phenoxyethanol, a preservative that has been shown to be safe when used in a 10-valent pneumococcal conjugate vaccine (18). Our study confirmed these findings with BE-PCV-14, with its similar safety profile as the comparator used in this study.

The immunogenicity to common serotypes between BE-PCV-14 and PCV-13 was comparable, and subjects in the BE-PCV-14 group elicited a robust response to serotypes 22F and 33F, the two new serotypes included in this vaccine.

Despite the reduction in IPD incidence after introducing PCVs in national immunization programs, there has been an insurgence of IPD cases caused by serotypes that are not included in the existing pneumococcal conjugate vaccines (19). In recent years, serotypes 22F and 33F have surfaced as the predominant causes of pneumococcal disease (PD) with associated mortality, morbidity, and antibiotic resistance in the pediatric population globally (20–22) (doi: 10.1016/j.vaccine.2018.05.026) (19, 23–25). BE-PCV-14 induced strong and robust immune responses with 88.6% and 67.1% of infants seroconverted against serotypes 22F and 33F, respectively. These results were further strengthened by another phase 3 study testing BE-PCV-14, conducted in a large cohort of infants, where 94.10% of infants were seroconverted against serotype 22F and 73.20% of infants were seroconverted against serotype 33F. When compared to the lowest-performing serotype from PCV-13, the immune responses in terms of seroconversion rates and GMC ratios were found to be non-inferior and superior, respectively. These results were in line with those of other 15-valent and 20-valent PCVs carrying these two serotypes (26). Developing pneumococcal vaccines incorporating broader and novel serotypes will alleviate the disease burden of IPD, especially emerging from non-vaccine serotypes.

Not only non-vaccine serotypes but some serotypes such as serotypes 3 and 19A included in the current PCVs still contribute to residual PD cases (27). Several studies have shown that there is only a limited effect of PCV-13 on reducing IPD cases caused by serotype 3 in children under 5 years of age ( (5, 8, 28, 29). Notably, 83.5% of BE-PCV-14-vaccinated children seroconverted against serotype 3 in the pivotal phase 3 study, and 76.5% seroconverted in the current study. Moreover, a stronger immune response was elicited by serotype 3 of BE-PCV-14 when compared to PCV-13. A similar trend was observed with a 15-valent PCV, where authors reported improved immunogenicity against serotype 3 compared to PCV-13 across pediatric and adult populations (30). Given that serotype 3 remains the primary cause of residual disease, even in individuals fully vaccinated with PCVs, caution is warranted in projecting PCV effectiveness solely based on the serotypes included in the vaccine. The superior immunogenicity induced by BE-PCV-14 and the 15-valent PCV against serotype 3 underscores the enhanced immunogenicity against challenging serotypes including serotypes 19A and 19F by newer PCVs.

We also assessed how effective BE-PCV-14 is in providing cross-protection against serotype 6A. Given the close structural resemblance between serotype 6A and serotype 6B ^24,25^, the latter of which is included in the BE-PCV-14 vaccine, we assessed if the vaccination would stimulate an immune response against both strains. There was more than a twofold rise in the GMC of anti-PnCPS IgG from pre-vaccination levels against serotype 6A, suggesting robust cross-protection. *Post hoc* analyses of GMC and seroconversion showed that protection against serotype 6A by BE-PCV-14 was non-inferior to that by PCV-13 (as per non-inferiority criteria outlined by the WHO). Importantly, a greater than sevenfold rise in serotype 6A OPA-specific functional antibody titers was observed in the pivotal phase 3 study (13). These data re-confirm that serotype 6B in BE-PCV-14 offers significant cross-protection to serotype 6A. Studies have shown a similar cross-protection profile for other pneumococcal vaccines, including the 23-valent polysaccharide vaccine (31–33).

While the pivotal phase 3 study showed the safety and immunogenicity of the single-dose presentation, we conducted this phase 3 trial in a smaller cohort of infants (n = 300) to evaluate the multi-dose presentation of BE-PCV-14. As demonstrated for the single-dose presentation, the BE-PCV-14 multi-dose presentation with 2-phenoxyethanol as a preservative was found to be safe, tolerable, and immunogenic. Multi-dose presentations reduce vaccine wastage, increase cost-effectiveness, and change logistics/cold chain factors (34). Vaccine manufacturing costs are lower for such multi-dose preparations, as it reduces filling costs, the need for overfill adjustments, and the cost of packaging (34). Biological E’s multi-dose presentation of BE-PCV-14 is likely to help expand access to target populations in need, particularly in the developing world.

This study is limited by its sample size of 300 infants with a primary objective of assessing the overall safety of the multi-dose presentation of BE-PCV-14. This particular study was not powered to assess immunogenicity in comparison to a licensed comparator. However, in the *post hoc* analysis, we demonstrated that all 14 serotypes of BE-PCV-14 met non-inferiority (NI) criteria in terms of GMC ratios. Moreover, our recently published pivotal phase 3 study of the single-dose preparation of BE-PCV-14 also showed non-inferiority to PCV-13 for all common and unique serotypes (13). Another limitation in both phase 3 studies was restricting the conduct of the studies in one geographic region. Similar studies in other regions of the world are being planned to expand the global use of this vaccine.

In conclusion, we show that Biological E’s multi-dose presentation of a pneumococcal conjugate vaccine with coverage against 14 serotypes is safe when administered to infants and elicits a strong and robust immune response. It also induced a cross-protective immune response to serotype 6A. The overall safety of this vaccine is further supported by data from a single-dose presentation of the vaccine that has been tested on over 3,000 individuals in multiple clinical trials. BE-PCV-14 is an important addition to the repertoire of present PCV vaccines.

## Data Availability

The raw data supporting the conclusions of this article will be made available by the authors, without undue reservation.
