# Supplementary material for: Immunogenicity and safety of a multi-human dose formulation of Biological E’s 14-valent pneumococcal polysaccharide conjugate vaccine (PNEUBEVAX 14®) administered to 6–8-week-old healthy infants: a phase 3, single-blind, randomized, active-controlled study
Source: Front Immunol. 2025 Apr 7;16:1550227. doi: 10.3389/fimmu.2025.1550227 (PMC12010144; doi:10.3389/fimmu.2025.1550227)
Supplement: Supplementary file 1 [file DataSheet1.docx]

**Supplementary information**

**Supplementary table 1: Summary of Vaccination Details - Safety Population (N=300)**

| **Vaccination** | **BE-PCV14 (N=150)** | **Prevenar13 (N=150)** | **Overall (N=300)** |
| --- | --- | --- | --- |
| BCG | 145 (96.7%) | 149 (99.3%) | 294 (98.0%) |
| Hepatitis-B | 150 (100.0%) | 150 (100.0%) | 300 (100.0%) |
| Polio^#^ | 150 (100.0%) | 150 (100.0%) | 300 (100.0%) |
| DTwP-HepB-Hib (Pentavalent)^#^ | 150 (100.0%) | 150 (100.0%) | 300 (100.0%) |
| **Note:**Percentage were calculated with respective column header count as denominator. N: Sample Size.  ^#^Concomitantly given along with PCV14 valent | | | |

**Supplementary table 2: Summary of Concomitant Medication - Safety Population (N=300)**

| **Therapeutic class / Generic Name, N1 (%)** | **BE-PCV14**  **(N=150)** | **Prevenar13**  **(N=150)** |
| --- | --- | --- |
| Visit 1 (Day 0) |  |  |
| Analgesics | 2 (1.33%) | 8 (5.33%) |
| Paracetamol | 2 (1.33%) | 8 (5.33%) |
| Visit 2 (Day 28) |  |  |
| Analgesics | 5 (3.33%) | 4 (2.67%) |
| Paracetamol | 5 (3.33%) | 4 (2.67%) |
| Visit 3 (Day 56) |  |  |
| Analgesics | 3 (2.00%) | 5 (3.33%) |
| Paracetamol | 3 (2.00%) | 5 (3.33%) |
| **Note:** Percentages were calculated using respective column header group count as denominator. n: Subject Count, N: Sample Size. | | |

**Supplementary table 3: Summary of AEs by SOC and PT by Causality–(N=300)**

| **SOC/PT/Causality**  **N1 (%) [95% CI] n** | **BE-PCV14 (N=150)** | **Prevenar 13 (N=150)** |
| --- | --- | --- |
| Overall | 35 (23.3%) [16.82:30.93] 66 | 35 (23.3%) [16.82:30.93] 71 |
| Gastrointestinal disorders | 0 (0.0%) [NE] 0 | 1 (0.7%) [0.02:3.66] 1 |
| Vomiting | 0 (0.0%) [NE] 0 | 1 (0.7%) [0.02:3.66] 1 |
| Certain | 0 (0.0%) [NE] 0 | 1 (0.7%) [0.02:3.66] 1 |
| Probable | 0 (0.0%) [NE] 0 | 0 (0.0%) [NE] 0 |
| Possible | 0 (0.0%) [NE] 0 | 0 (0.0%) [NE] 0 |
| Unlikely | 0 (0.0%) [NE] 0 | 0 (0.0%) [NE] 0 |
| Unrelated | 0 (0.0%) [NE] 0 | 0 (0.0%) [NE] 0 |
| Unclassifiable | 0 (0.0%) [NE] 0 | 0 (0.0%) [NE] 0 |
| General disorders and administration site conditions | 35 (23.3%) [16.82:30.93] 66 | 35 (23.3%) [16.82:30.93] 70 |
| Injection site erythema | 10 (6.7%) [3.24:11.92] 10 | 7 (4.7%) [1.90:9.38] 7 |
| Certain | 0 (0.0%) [NE] 0 | 0 (0.0%) [NE] 0 |
| Probable | 10 (6.7%) [3.24:11.92] 10 | 7 (4.7%) [1.90:9.38] 7 |
| Possible | 0 (0.0%) [NE] 0 | 0 (0.0%) [NE] 0 |
| Unlikely | 0 (0.0%) [NE] 0 | 0 (0.0%) [NE] 0 |
| Unrelated | 0 (0.0%) [NE] 0 | 0 (0.0%) [NE] 0 |
| Unclassifiable | 0 (0.0%) [NE] 0 | 0 (0.0%) [NE] 0 |
| Injection site induration | 1 (0.7%) [0.02:3.66] 1 | 0 (0.0%) [NE] 0 |
| Certain | 1 (0.7%) [0.02:3.66] 1 | 0 (0.0%) [NE] 0 |
| Probable | 0 (0.0%) [NE] 0 | 0 (0.0%) [NE] 0 |
| Possible | 0 (0.0%) [NE] 0 | 0 (0.0%) [NE] 0 |
| Unlikely | 0 (0.0%) [NE] 0 | 0 (0.0%) [NE] 0 |
| Unrelated | 0 (0.0%) [NE] 0 | 0 (0.0%) [NE] 0 |
| Unclassifiable | 0 (0.0%) [NE] 0 | 0 (0.0%) [NE] 0 |
| Injection site pain | 17 (11.3%) [6.74:17.52] 24 | 19 (12.7%) [7.80:19.07] 25 |
| Certain | 11 (7.3%) [3.72:12.74] 11 | 18 (12.0%) [7.27:18.30] 23 |
| Probable | 6 (4.0%) [1.48:8.50] 13 | 1 (0.7%) [0.02:3.66] 2 |
| Possible | 0 (0.0%) [NE] 0 | 0 (0.0%) [NE] 0 |
| Unlikely | 0 (0.0%) [NE] 0 | 0 (0.0%) [NE] 0 |
| Unrelated | 0 (0.0%) [NE] 0 | 0 (0.0%) [NE] 0 |
| Unclassifiable | 0 (0.0%) [NE] 0 | 0 (0.0%) [NE] 0 |
| Injection site swelling | 10 (6.7%) [3.24:11.92] 14 | 13 (8.7%) [4.70:14.36] 16 |
| Certain | 0 (0.0%) [NE] 0 | 5 (3.3%) [1.09:7.61] 7 |
| Probable | 10 (6.7%) [3.24:11.92] 14 | 8 (5.3%) [2.33:10.24] 9 |
| Possible | 0 (0.0%) [NE] 0 | 0 (0.0%) [NE] 0 |
| Unlikely | 0 (0.0%) [NE] 0 | 0 (0.0%) [NE] 0 |
| Unrelated | 0 (0.0%) [NE] 0 | 0 (0.0%) [NE] 0 |
| Unclassifiable | 0 (0.0%) [NE] 0 | 0 (0.0%) [NE] 0 |
| Irritability Postvaccinal | 5 (3.3%) [1.09:7.61] 12 | 6 (4.0%) [1.48:8.50] 9 |
| Certain | 0 (0.0%) [NE] 0 | 2 (1.3%) [0.16:4.73] 3 |
| Probable | 5 (3.3%) [1.09:7.61] 12 | 5 (3.3%) [1.09:7.61] 6 |
| Possible | 0 (0.0%) [NE] 0 | 0 (0.0%) [NE] 0 |
| Unlikely | 0 (0.0%) [NE] 0 | 0 (0.0%) [NE] 0 |
| Unrelated | 0 (0.0%) [NE] 0 | 0 (0.0%) [NE] 0 |
| Unclassifiable | 0 (0.0%) [NE] 0 | 0 (0.0%) [NE] 0 |
| Pyrexia | 5 (3.3%) [1.09:7.61] 5 | 12 (8.0%) [4.20:13.56] 13 |
| Certain | 3 (2.0%) [0.41:5.73] 3 | 2 (1.3%) [0.16:4.73] 2 |
| Probable | 0 (0.0%) [NE] 0 | 5 (3.3%) [1.09:7.61] 6 |
| Possible | 2 (1.3%) [0.16:4.73] 2 | 5 (3.3%) [1.09:7.61] 5 |
| Unlikely | 0 (0.0%) [NE] 0 | 0 (0.0%) [NE] 0 |
| Unrelated | 0 (0.0%) [NE] 0 | 0 (0.0%) [NE] 0 |
| Unclassifiable | 0 (0.0%) [NE] 0 | 0 (0.0%) [NE] 0 |
| **Note:** Percentages were calculated using column header group count as denominator. 95% CI were calculated by Clopper -Pearson Method. N1: Subject Count, N: Sample Size, n: Event Count **General Note:** • All AE's were represented as: Subject count (Percentage of subjects) [95% CI] Event Count. | | |

Supplementary table 4: Summary of Local AEs by SOC & PT– Safety Population (N=300)

| **SOC/PT**  **N1 (%) [95% CI] n** | **BE-PCV14 (N=150)** | **Prevenar 13 (N=150)** |
| --- | --- | --- |
| Overall | 30 (20.0%) [13.92:27.30] 49 | 30 (20.0%) [13.92:27.30] 48 |
| General Disorders And Administration Site Conditions | 30 (20.0%) [13.92:27.30] 49 | 30 (20.0%) [13.92:27.30] 48 |
| Injection Site Erythema | 10 (6.7%) [3.24:11.92] 10 | 7 (4.7%) [1.90:9.38] 7 |
| Injection Site Induration | 1 (0.7%) [0.02:3.66] 1 | 0 (0.0%) [NE] 0 |
| Injection Site Pain | 17 (11.3%) [6.74:17.52] 24 | 19 (12.7%) [7.80:19.07] 25 |
| Injection Site Swelling | 10 (6.7%) [3.24:11.92] 14 | 13 (8.7%) [4.70:14.36] 16 |
| \| **Note:** Percentages were calculated using column header count as denominator. 95% CI were calculated by Clopper -Pearson Method.  N1: Subject Count, N: Sample Size, n: Event Count **General Note:** •   All AE's were represented as: Subject count (Percentage of subjects) [95% CI] Event Count. \| \| --- \| | | |

**Supplementary immunogenicity data tables**

**Summary of serotype specific anti-PnCPS IgG antibody concentration – PP Population (N=297)**

| **Anti-PnCPS IgG antibody concentration, N1 (%) 95% CI** | **BE-PCV14 (N=149)** | **Prevenar13 (N=148)** |
| --- | --- | --- |
| **Serotype 1** |  |  |
| **Pre Vaccination (Day 0)** |  |  |
| Seroconverted | 71 (47.7%) | 58 (39.2%) |
|  | [39.41 : 55.98] | [31.28 : 47.54] |
| Not Seroconverted | 78 (52.3%) | 90 (60.8%) |
|  | [44.02 : 60.59] | [52.46 : 68.72] |
| **Post Vaccination (Day 86)** |  |  |
| Seroconverted | 141 (94.6%) | 146 (98.6%) |
|  | [89.69 : 97.65] | [95.20 : 99.84] |
| Not Seroconverted | 8 (5.4%) | 2 (1.4%) |
|  | [2.35 : 10.31] | [0.16 : 4.80] |
| **Serotype 3** |  |  |
| **Pre Vaccination (Day 0)** |  |  |
| Seroconverted | 51 (34.2%) | 44 (29.7%) |
|  | [26.66 : 42.44] | [22.50 : 37.79] |
| Not Seroconverted | 98 (65.8%) | 104 (70.3%) |
|  | [57.56 : 73.34] | [62.21 : 77.50] |
| **Post Vaccination (Day 86)** |  |  |
| Seroconverted | 114 (76.5%) | 119 (80.4%) |
|  | [68.88 : 83.06] | [73.09 : 86.47] |
| Not Seroconverted | 35 (23.5%) | 29 (19.6%) |
|  | [16.94 : 31.12] | [13.53 : 26.91] |
| **Serotype 4** |  |  |
| **Pre Vaccination (Day 0)** |  |  |
| Seroconverted | 106 (71.1%) | 84 (56.8%) |
|  | [63.16 : 78.26] | [48.37 : 64.87] |
| Not Seroconverted | 43 (28.9%) | 64 (43.2%) |
|  | [21.74 : 36.84] | [35.13 : 51.63] |
| **Post Vaccination (Day 86)** |  |  |
| Seroconverted | 136 (91.3%) | 142 (95.9%) |
|  | [85.54 : 95.27] | [91.39 : 98.50] |
| Not Seroconverted | 13 (8.7%) | 6 (4.1%) |
|  | [4.73 : 14.46] | [1.50 : 8.61] |
| **Serotype 5** |  |  |
| **Pre Vaccination (Day 0)** |  |  |
| Seroconverted | 65 (43.6%) | 56 (37.8%) |
|  | [35.53 : 51.98] | [30.00 : 46.17] |
| Not Seroconverted | 84(56.4%) | 92(62.2%) |
|  | [48.02 : 64.47] | [53.83 : 70.00] |
| **Post Vaccination (Day 86)** |  |  |
| Seroconverted | 132 (88.6%) | 128 (86.5%) |
|  | [82.36 : 93.21] | [79.90 : 91.55] |
| Not Seroconverted | 17 (11.4%) | 20 (13.5%) |
|  | [6.79 : 17.64] | [8.45 : 20.10] |
| **Serotype 6B** |  |  |
| **Pre Vaccination (Day 0)** |  |  |
| Seroconverted | 72 (48.3%) | 66 (44.6%)  [36.43 : 52.98] |
|  | [40.07 : 56.64] |  |
| Not Seroconverted | 77 (51.7%) | 82 (55.4%)  [47.02 : 63.57] |
|  | [43.36 : 59.93] |  |
| **Post Vaccination (Day 86)** |  |  |
| Seroconverted | 121 (81.2%) | 108 (73.0%)  [65.06 : 79.94] |
|  | [74.00 : 87.13] |  |
| Not Seroconverted | 28 (18.8%) | 40 (27.0%)  [20.06 : 34.94] |
|  | [12.87 : 26.00] |  |
| **Serotype 7F** |  |  |
| **Pre Vaccination (Day 0)** |  |  |
| Seroconverted | 83 (55.7%) | 67 (45.3%) |
|  | [47.35 : 63.83] | [37.08 : 53.65] |
| Not Seroconverted | 66 (44.3%) | 81 (54.7%) |
|  | [36.17 : 52.65] | [46.35 : 62.92] |
| **Post Vaccination (Day 86)** |  |  |
| Seroconverted | 144 (96.6%) | 144 (97.3%) |
|  | [92.34 : 98.90] | [93.22 : 99.26] |
| Not Seroconverted | 5 (3.4%) | 4 (2.7%) |
|  | [1.10 : 7.66] | [0.74 : 6.78] |
| **Serotype 9V** |  |  |
| **Pre Vaccination (Day 0)** |  |  |
| Seroconverted | 87(58.4%) | 74(50.0%) |
|  | [50.04 : 66.40] | [41.68 : 58.32] |
| Not Seroconverted | 62(41.6%) | 74(50.0%) |
|  | [33.60 : 49.96] | [41.68 : 58.32] |
| **Post Vaccination (Day 86)** |  |  |
| Seroconverted | 141(94.6%) | 132(89.2%) |
|  | [89.69 : 97.65] | [83.04 : 93.69] |
| Not Seroconverted | 8(5.4%) | 16(10.8%) |
|  | [2.35 : 10.31] | [6.31 : 16.96] |
| **Serotype 14** |  |  |
| **Pre Vaccination (Day 0)** |  |  |
| Seroconverted | 147(98.7%) | 147(99.3%) |
|  | [95.24 : 99.84] | [96.29 : 99.98] |
| Not Seroconverted | 2(1.3%) | 1(0.7%) |
|  | [0.16 : 4.76] | [0.02 : 3.71] |
| **Post Vaccination (Day 86)** |  |  |
| Seroconverted | 149(100.0%) | 148(100.0%) |
|  | [97.55 : 100.00] | [97.54 : 100.00] |
| Not Seroconverted | 0(0.0%) | 0(0.0%) |
|  | [0.00 : 2.45] | [0.00 : 2.46] |
| **Serotype 18C** |  |  |
| **Pre Vaccination (Day 0)** |  |  |
| Seroconverted | 127(85.2%) | 117(79.1%) |
|  | [78.50 : 90.51] | [71.61 : 85.30] |
| Not Seroconverted | 22(14.8%) | 31(20.9%) |
|  | [9.49 : 21.50] | [14.70 : 28.39] |
| **Post Vaccination (Day 86)** |  |  |
| Seroconverted | 136(91.3%) | 141(95.3%) |
|  | [85.54 : 95.27] | [90.50 : 98.08] |
| Not Seroconverted | 13(8.7%) | 7(4.7%) |
|  | [4.73 : 14.46] | [1.92 : 9.50] |
| **Serotype 19A** |  |  |
| **Pre Vaccination (Day 0)** |  |  |
| Seroconverted | 148(99.3%) | 145(98.0%) |
|  | [96.32 : 99.98] | [94.19 : 99.58] |
| Not Seroconverted | 1(0.7%) | 3(2.0%) |
|  | [0.02 : 3.68] | [0.42 : 5.81] |
| **Post Vaccination (Day 86)** |  |  |
| Seroconverted | 149(100.0%) | 148(100.0%) |
|  | [97.55 : 100.00] | [97.54 : 100.00] |
| Not Seroconverted | 0(0.0%) | 0(0.0%) |
|  | [0.00 : 2.45] | [0.00 : 2.46] |
| **Serotype 19F** |  |  |
| **Pre Vaccination (Day 0)** |  |  |
| Seroconverted | 124(83.2%) | 107(72.3%) |
|  | [76.24 : 88.84] | [64.35 : 79.33] |
| Not Seroconverted | 25(16.8%) | 41(27.7%) |
|  | [11.16 : 23.76] | [20.67 : 35.65] |
| **Post Vaccination (Day 86)** |  |  |
| Seroconverted | 145(97.3%) | 145(98.0%) |
|  | [93.27 : 99.26] | [94.19 : 99.58] |
| Not Seroconverted | 4(2.7%) | 3(2.0%) |
|  | [0.74 : 6.73] | [0.42 : 5.81] |
| **Serotype 23F** |  |  |
| **Pre Vaccination (Day 0)** |  |  |
| Seroconverted | 98(65.8%) | 80(54.1%) |
|  | [57.56 : 73.34] | [45.68 : 62.27] |
| Not Seroconverted | 51(34.2%) | 68(45.9%) |
|  | [26.66 : 42.44] | [37.73 : 54.32] |
| **Post Vaccination (Day 86)** |  |  |
| Seroconverted | 129(86.6%) | 114(77.0%) |
|  | [80.03 : 91.60] | [69.40 : 83.54] |
| Not Seroconverted | 20(13.4%) | 34(23.0%) |
|  | [8.40 : 19.97] | [16.46 : 30.60] |
| **Note:** Percentages were calculated using column header count as denominator. 95% CI were calculated by Clopper -Pearson Method. N1: Subject Count, N: Sample Size. | | |

**Summary of Geometric Mean Concentrations (GMCs) of serotype specific anti-PnCPS IgG antibodies – PP Population (N=297).**

| **Anti-PnCPS IgG antibody concentration** | **BE-PCV14 (N=149)** | **Prevenar13 (N=148)** |
| --- | --- | --- |
| **Serotype 1** |  |  |
| **Pre-Vaccination (Day 0)** |  |  |
| N_1_ | 149 | 148 |
| GMC | 0.31 | 0.27 |
| 95 % CI of GMC | (0.28:0.35) | (0.24:0.32) |
| Median | 0.32 | 0.27 |
| (Q1:Q3) | (0.15:0.63) | (0.14:0.57) |
| Range (Min.:Max.) | (0.03:3.02) | (0.00:5.14) |
| **Post-Vaccination (Day 86)** |  |  |
| N_1_ | 149 | 148 |
| GMC | 2.06 | 2.30 |
| 95 % CI of GMC | (1.77:2.39) | (2.03:2.59) |
| Median | 1.71 | 2.42 |
| (Q1:Q3) | (1.03:4.38) | (1.39:3.89) |
| Range (Min.:Max.) | (0.10:71.46) | (0.12:46.91) |
| GMCR (Post GMC/Pre GMC) | 6.65 | 8.52 |
| **Serotype 3** |  |  |
| **Pre-Vaccination (Day 0)** |  |  |
| N_1_ | 149 | 148 |
| GMC | 0.24 | 0.20 |
| 95 % CI of GMC | (0.22:0.28) | (0.17:0.22) |
| Median | 0.23 | 0.17 |
| (Q1:Q3) | (0.13:0.53) | (0.10:0.42) |
| Range (Min.:Max.) | (0.03:1.52) | (0.02:1.60) |
| **Post-Vaccination (Day 86)** |  |  |
| N_1_ | 149 | 148 |
| GMC | 0.61 | 0.58 |
| 95 % CI of GMC | (0.55:0.67) | (0.52:0.63) |
| Median | 0.59 | 0.60 |
| (Q1:Q3) | (0.36:0.91) | (0.41:0.86) |
| Range (Min.:Max.) | (0.06:3.00) | (0.05:2.66) |
| GMCR (Post GMC/Pre GMC) | 2.54 | 2.90 |
| **Serotype 4** |  |  |
| **Pre-Vaccination (Day 0)** |  |  |
| N_1_ | 149 | 148 |
| GMC | 0.56 | 0.47 |
| 95 % CI of GMC | (0.50:0.63) | (0.42:0.53) |
| Median | 0.61 | 0.39 |
| (Q1:Q3) | (0.32:1.07) | (0.27:0.92) |
| Range (Min.:Max.) | (0.06:3.80) | (0.04:3.68) |
| **Post-Vaccination (Day 86)** |  |  |
| N_1_ | 149 | 148 |
| GMC | 1.85 | 2.05 |
| 95 % CI of GMC | (1.60:2.15) | (1.81:2.34) |
| Median | 1.94 | 2.33 |
| (Q1:Q3) | (0.87:3.79) | (1.12:3.62) |
| Range (Min.:Max.) | (0.09:15.95) | (0.09:14.69) |
| GMCR (Post GMC/Pre GMC) | 3.30 | 4.36 |
| **Serotype 5** |  |  |
| **Pre-Vaccination (Day 0)** |  |  |
| N_1_ | 149 | 148 |
| GMC | 0.27 | 0.26 |
| 95 % CI of GMC | (0.24:0.32) | (0.23:0.30) |
| Median | 0.27 | 0.25 |
| (Q1:Q3) | (0.13:0.60) | (0.11:0.58) |
| Range (Min.:Max.) | (0.00:2.41) | (0.04:1.95) |
| **Post-Vaccination (Day 86)** |  |  |
| N_1_ | 149 | 148 |
| GMC | 1.48 | 1.30 |
| 95 % CI of GMC | (1.28:1.72) | (1.10:1.52) |
| Median | 1.44 | 1.33 |
| (Q1:Q3) | (0.69:3.30) | (0.67:2.80) |
| Range (Min.:Max.) | (0.04:23.06) | (0.03:21.18) |
| GMCR (Post GMC/Pre GMC) | 5.48 | 5.00 |
| **Serotype 6B** |  |  |
| **Pre-Vaccination (Day 0)** |  |  |
| N_1_ | 149 | 148 |
| GMC | 0.35 | 0.25 |
| 95 % CI of GMC | (0.29:0.41) | (0.19:0.31) |
| Median | 0.33 | 0.30 |
| (Q1:Q3) | (0.14:0.86) | (0.12:0.73) |
| Range (Min.:Max.) | (0.00:9.29) | (0.00:5.85) |
| **Post-Vaccination (Day 86)** |  |  |
| N_1_ | 149 | 148 |
| GMC | 1.88 | 1.35 |
| 95 % CI of GMC | (1.49:2.36) | (1.08:1.70) |
| Median | 1.97 | 1.67 |
| (Q1:Q3) | (0.64:7.56) | (0.30:4.98) |
| Range (Min.:Max.) | (0.04:30.23) | (0.03:34.79) |
| GMCR (Post GMC/Pre GMC) | 5.37 | 5.40 |
| **Serotype 7F** |  |  |
| **Pre-Vaccination (Day 0)** |  |  |
| N_1_ | 149 | 148 |
| GMC | 0.42 | 0.26 |
| 95 % CI of GMC | (0.36:0.49) | (0.20:0.32) |
| Median | 0.42 | 0.28 |
| (Q1:Q3) | (0.19:0.88) | (0.12:0.78) |
| Range (Min.:Max.) | (0.04:7.77) | (0.00:3.74) |
| **Post-Vaccination (Day 86)** |  |  |
| N_1_ | 149 | 148 |
| GMC | 2.79 | 3.34 |
| 95 % CI of GMC | (2.40:3.23) | (2.93:3.81) |
| Median | 2.99 | 3.53 |
| (Q1:Q3) | (1.59:4.84) | (1.96:6.67) |
| Range (Min.:Max.) | (0.20:199.22) | (0.12:34.23) |
| GMCR (Post GMC/Pre GMC) | 6.64 | 12.85 |
| **Serotype 9V** |  |  |
| **Pre-Vaccination (Day 0)** |  |  |
| N_1_ | 149 | 148 |
| GMC | 0.45 | 0.35 |
| 95 % CI of GMC | (0.37:0.54) | (0.29:0.42) |
| Median | 0.50 | 0.34 |
| (Q1:Q3) | (0.18:1.11) | (0.17:0.93) |
| Range (Min.:Max.) | (0.00:4.97) | (0.00:4.97) |
| **Post-Vaccination (Day 86)** |  |  |
| N_1_ | 149 | 148 |
| GMC | 2.33 | 1.82 |
| 95 % CI of GMC | (1.98:2.74) | (1.54:2.15) |
| Median | 2.17 | 2.14 |
| (Q1:Q3) | (1.08:4.59) | (0.81:3.71) |
| Range (Min.:Max.) | (0.05:38.96) | (0.06:34.16) |
| GMCR (Post GMC/Pre GMC) | 5.18 | 5.20 |
| **Serotype 14** |  |  |
| **Pre-Vaccination (Day 0)** |  |  |
| N_1_ | 149 | 148 |
| GMC | 6.09 | 5.32 |
| 95 % CI of GMC | (5.39:6.90) | (4.71:6.00) |
| Median | 6.29 | 5.01 |
| (Q1:Q3) | (3.58:12.25) | (2.81:10.88) |
| Range (Min.:Max.) | (0.19:35.26) | (0.30:64.98) |
| **Post-Vaccination (Day 86)** |  |  |
| N_1_ | 149 | 148 |
| GMC | 7.93 | 6.22 |
| 95 % CI of GMC | (6.87:9.16) | (5.34:7.24) |
| Median | 8.64 | 5.71 |
| (Q1:Q3) | (2.85:18.23) | (2.17:16.77) |
| Range (Min.:Max.) | (0.78:79.91) | (0.68:77.68) |
| GMCR (Post GMC/Pre GMC) | 1.30 | 1.17 |
| **Serotype 18C** |  |  |
| **Pre-Vaccination (Day 0)** |  |  |
| N_1_ | 149 | 148 |
| GMC | 1.01 | 0.83 |
| 95 % CI of GMC | (0.89:1.15) | (0.73:0.94) |
| Median | 1.02 | 0.78 |
| (Q1:Q3) | (0.45:2.06) | (0.40:1.78) |
| Range (Min.:Max.) | (0.13:7.85) | (0.05:10.30) |
| **Post-Vaccination (Day 86)** |  |  |
| N_1_ | 149 | 148 |
| GMC | 2.01 | 1.84 |
| 95 % CI of GMC | (1.71:2.36) | (1.60:2.10) |
| Median | 1.90 | 1.92 |
| (Q1:Q3) | (0.96:4.46) | (0.97:3.61) |
| Range (Min.:Max.) | (0.11:29.97) | (0.21:27.97) |
| GMCR (Post GMC/Pre GMC) | 1.99 | 2.22 |
| **Serotype 19A** |  |  |
| **Pre-Vaccination (Day 0)** |  |  |
| N_1_ | 149 | 148 |
| GMC | 2.14 | 1.83 |
| 95 % CI of GMC | (1.90:2.41) | (1.63:2.06) |
| Median | 2.06 | 1.93 |
| (Q1:Q3) | (1.02:4.08) | (0.93:3.34) |
| Range (Min.:Max.) | (0.29:15.39) | (0.15:20.11) |
| **Post-Vaccination (Day 86)** |  |  |
| N_1_ | 149 | 148 |
| GMC | 4.14 | 3.65 |
| 95 % CI of GMC | (3.59:4.78) | (3.19:4.17) |
| Median | 3.55 | 3.57 |
| (Q1:Q3) | (1.88:8.30) | (1.96:6.63) |
| Range (Min.:Max.) | (0.48:49.29) | (0.36:38.05) |
| GMCR (Post GMC/Pre GMC) | 1.93 | 1.99 |
| **Serotype 19F** |  |  |
| **Pre-Vaccination (Day 0)** |  |  |
| N_1_ | 149 | 148 |
| GMC | 1.04 | 0.91 |
| 95 % CI of GMC | (0.90:1.20) | (0.78:1.07) |
| Median | 1.12 | 1.03 |
| (Q1:Q3) | (0.45:2.19) | (0.30:2.17) |
| Range (Min.:Max.) | (0.09:11.86) | (0.11:20.52) |
| **Post-Vaccination (Day 86)** |  |  |
| N_1_ | 149 | 148 |
| GMC | 3.51 | 3.90 |
| 95 % CI of GMC | (3.00:4.11) | (3.36:4.51) |
| Median | 3.71 | 4.27 |
| (Q1:Q3) | (1.46:7.94) | (1.87:8.10) |
| Range (Min.:Max.) | (0.23:51.16) | (0.16:45.84) |
| GMCR (Post GMC/Pre GMC) | 3.38 | 4.29 |
| **Serotype 23F** |  |  |
| **Pre-Vaccination (Day 0)** |  |  |
| N_1_ | 149 | 148 |
| GMC | 0.53 | 0.41 |
| 95 % CI of GMC | (0.45:0.63) | (0.34:0.48) |
| Median | 0.59 | 0.44 |
| (Q1:Q3) | (0.19:1.36) | (0.15:1.03) |
| Range (Min.:Max.) | (0.04:7.91) | (0.03:13.84) |
| **Post-Vaccination (Day 86)** |  |  |
| N_1_ | 149 | 148 |
| GMC | 1.82 | 1.52 |
| 95 % CI of GMC | (1.48:2.24) | (1.24:1.88) |
| Median | 1.70 | 1.64 |
| (Q1:Q3) | (0.55:5.51) | (0.37:5.71) |
| Range (Min.:Max.) | (0.08:38.81) | (0.06:32.38) |
| GMCR (Post GMC/Pre GMC) | 3.43 | 3.71 |
| **Note:** N_1_: Subject Count, N: Sample Size GMCR = Geometric Mean Concentration Ratio; GMC = Geometric Mean Concentration | | |

**Proportion of subjects achieving >=2-fold, >=4-fold, GMFR & ≥4-fold rise in subjects already baseline seroconverted rise in anti-PnCPS IgG antibody concentrations – PP Population (N=297)**

| **Anti-PnCPS IgG against each of the vaccine serotype**  **N1 (%) [95% CI]** | **BE-PCV14 (N=149)** | **Prevenar13 (N=148)** |
| --- | --- | --- |
| **Serotype 1** |  |  |
| **Post Vaccination (Day 86)** |  |  |
| >=2 fold increase in all subjects | 128 (85.9%) [79.27:91.06] | 130 (87.8%) [81.46:92.63] |
| >=4 fold increase in all subjects | 102 (68.5%) [60.35:75.82] | 111 (75.0%) [67.22:81.75] |
| GMFR | 6.61 [5.49:7.96] | 8.36 [6.79:10.29] |
| Subjects who were Seroconverted (>=0.35 µg/mL) at baseline | 71 (47.7%) [39.41:55.98] | 58 (39.2%) [31.28:47.54] |
| >=4 fold increase | 37 (24.8%) [18.13:32.57] | 28 (18.9%) [12.95:26.17] |
| **Serotype 3** |  |  |
| **Post Vaccination (Day 86)** |  |  |
| >=2 fold increase in all subjects | 81 (54.4%) [46.01:62.54] | 93 (62.8%) [54.52:70.63] |
| >=4 fold increase in all subjects | 43 (28.9%) [21.74:36.84] | 62 (41.9%) [33.84:50.27] |
| GMFR | 2.50 [2.09:3.00] | 2.93 [2.45:3.51] |
| Subjects who were Seroconverted (>=0.35 µg/mL) at baseline | 51 (34.2%) [26.66:42.44] | 44 (29.7%) [22.50:37.79] |
| >=4 fold increase | 1 (0.7%) [0.02:3.68] | 1 (0.7%) [0.02:3.71] |
| **Serotype 4** |  |  |
| **Post Vaccination (Day 86)** |  |  |
| >=2 fold increase in all subjects | 101 (67.8%) [59.65:75.20] | 111 (75.0%) [67.22:81.75] |
| >=4 fold increase in all subjects | 69 (46.3%) [38.11:54.65] | 86 (58.1%) [49.73:66.16] |
| GMFR | 3.31 [2.72:4.03] | 4.33 [3.57:5.26] |
| Subjects who were Seroconverted (>=0.35 µg/mL) at baseline | 106 (71.1%) [63.16:78.26] | 84 (56.8%) [48.37:64.87] |
| >=4 fold increase | 37 (24.8%) [18.13:32.57] | 35 (23.6%) [17.06:31.32] |
| **Serotype 5** |  |  |
| **Post Vaccination (Day 86)** |  |  |
| >=2 fold increase in all subjects | 124 (83.2%) [76.24:88.84] | 117 (79.1%) [71.61:85.30] |
| >=4 fold increase in all subjects | 87 (58.4%) [50.04:66.40] | 83 (56.1%) [47.69:64.22] |
| GMFR | 5.41 [4.42:6.61] | 4.91 [4.03:5.99] |
| Subjects who were Seroconverted (>=0.35 µg/mL) at baseline | 65 (43.6%) [35.53:51.98] | 56 (37.8%) [30.00:46.17] |
| >=4 fold increase | 26 (17.4%) [11.73:24.51] | 20 (13.5%) [8.45:20.10] |
| **Serotype 6B** |  |  |
| **Post Vaccination (Day 86)** |  |  |
| >=2 fold increase in all subjects | 105 (70.5%) [62.45:77.65] | 95 (64.2%) [55.90:71.89] |
| >=4 fold increase in all subjects | 80 (53.7%) [45.35:61.89] | 78 (52.7%) [44.34:60.96] |
| GMFR | 5.44 [3.93:7.53] | 5.49 [3.78:7.96] |
| Subjects who were Seroconverted (>=0.35 µg/mL) at baseline | 72 (48.3%) [40.07:56.64] | 66 (44.6%) [36.43:52.98] |
| >=4 fold increase | 27 (18.1%) [12.29:25.26] | 29 (19.6%) [13.53:26.91] |
| **Serotype 7F** |  |  |
| **Post Vaccination (Day 86)** |  |  |
| >=2 fold increase in all subjects | 121 (81.2%) [74.00:87.13] | 133 (89.9%) [83.83:94.22] |
| >=4 fold increase in all subjects | 97 (65.1%) [56.87:72.72] | 116 (78.4%) [70.87:84.72] |
| GMFR | 6.59 [5.26:8.27] | 13.09 [9.90:17.30] |
| Subjects who were Seroconverted (>=0.35 µg/mL) at baseline | 83 (55.7%) [47.35:63.83] | 67 (45.3%) [37.08:53.65] |
| >=4 fold increase | 41 (27.5%) [20.53:35.43] | 42 (28.4%) [21.28:36.36] |
| **Serotype 9V** |  |  |
| **Post Vaccination (Day 86)** |  |  |
| >=2 fold increase in all subjects | 102 (68.5%) [60.35:75.82] | 113 (76.4%) [68.68:82.94] |
| >=4 fold increase in all subjects | 80 (53.7%) [45.35:61.89] | 84 (56.8%) [48.37:64.87] |
| GMFR | 5.21 [4.02:6.76] | 5.23 [4.06:6.73] |
| Subjects who were Seroconverted (>=0.35 µg/mL) at baseline | 87 (58.4%) [50.04:66.40] | 74 (50.0%) [41.68:58.32] |
| >=4 fold increase | 30 (20.1%) [14.02:27.48] | 25 (16.9%) [11.24:23.92] |
| **Serotype 14** |  |  |
| Post Vaccination (Day 86) |  |  |
| >=2 fold increase in all subjects | 55 (36.9%) [29.16:45.20] | 52 (35.1%) [27.48:43.40] |
| >=4 fold increase in all subjects | 23 (15.4%) [10.04:22.26] | 27 (18.2%) [12.38:25.42] |
| GMFR | 1.30 [1.07:1.58] | 1.17 [0.96:1.42] |
| Subjects who were Seroconverted (>=0.35 µg/mL) at baseline | 147 (98.7%) [95.24:99.84] | 147 (99.3%) [96.29:99.98] |
| >=4 fold increase | 21 (14.1%) [8.94:20.73] | 26 (17.6%) [11.81:24.67] |
| **Serotype 18C** |  |  |
| **Post Vaccination (Day 86)** |  |  |
| >=2 fold increase in all subjects | 79 (53.0%) [44.68:61.24] | 80 (54.1%) [45.68:62.27] |
| >=4 fold increase in all subjects | 39 (26.2%) [19.32:34.00] | 40 (27.0%) [20.06:34.94] |
| GMFR | 1.99 [1.68:2.36] | 2.21 [1.88:2.59] |
| Subjects who were Seroconverted (>=0.35 µg/mL) at baseline | 127 (85.2%) [78.50:90.51] | 117 (79.1%) [71.61:85.30] |
| >=4 fold increase | 26 (17.4%) [11.73:24.51] | 18 (12.2%) [7.37:18.54] |
| **Serotype 19A** |  |  |
| Post Vaccination (Day 86) |  |  |
| >=2 fold increase in all subjects | 79 (53.0%) [44.68:61.24] | 74 (50.0%) [41.68:58.32] |
| >=4 fold increase in all subjects | 33 (22.1%) [15.76:29.67] | 44 (29.7%) [22.50:37.79] |
| GMFR | 1.93 [1.64:2.29] | 1.99 [1.67:2.37] |
| Subjects who were Seroconverted (>=0.35 µg/mL) at baseline | 148 (99.3%) [96.32:99.98] | 145 (98.0%) [94.19:99.58] |
| >=4 fold increase | 32 (21.5%) [15.18:28.94] | 42 (28.4%) [21.28:36.36] |
| **Serotype 19F** |  |  |
| Post Vaccination (Day 86) |  |  |
| >=2 fold increase in all subjects | 94 (63.1%) [54.80:70.84] | 105 (70.9%) [62.92:78.11] |
| >=4 fold increase in all subjects | 71 (47.7%) [39.41:55.98] | 75 (50.7%) [42.34:58.98] |
| GMFR | 3.37 [2.68:4.25] | 4.27 [3.35:5.46] |
| Subjects who were Seroconverted (>=0.35 µg/mL) at baseline | 124 (83.2%) [76.24:88.84] | 107 (72.3%) [64.35:79.33] |
| >=4 fold increase | 46 (30.9%) [23.57:38.95] | 39 (26.4%) [19.46:34.22] |
| **Serotype 23F** |  |  |
| **Post Vaccination (Day 86)** |  |  |
| >=2 fold increase in all subjects | 89 (59.7%) [51.39:67.68] | 94 (63.5%) [55.21:71.26] |
| >=4 fold increase in all subjects | 69 (46.3%) [38.11:54.65] | 76 (51.4%) [43.01:59.64] |
| GMFR | 3.41 [2.59:4.49] | 3.76 [2.85:4.97] |
| Subjects who were Seroconverted (>=0.35 µg/mL) at baseline | 98 (65.8%) [57.56:73.34] | 80 (54.1%) [45.68:62.27] |
| >=4 fold increase | 34 (22.8%) [16.35:30.40] | 29 (19.6%) [13.53:26.91] |
| **Note:** Percentage was calculated using respective header count as denominator 95% CI for was calculated using Clopper Pearson Method | | |

**Proportion of subjects achieving fold rise in geometric mean concentration above pre-vaccination levels in Serotype 6A – PP Population (N=297)**

| **Anti-PnCPS IgG antibody concentration** | **BE-PCV14 (N=149)**  **Prevac (Day 0)** | **BE-PCV14 (N=148)**  **Postvac (Day 86)** | **Fold Rise**  **Postvac / Prevac** |
| --- | --- | --- | --- |
| **Serotype 6A** |  |  |  |
| GMC | 0.48 | 0.98 | **2.04** |
| **Note:** N: Sample Size GMC = Geometric Mean Concentration | | |  |

**Seroconversion of IgG antibodies against serotypes 22F and 33F in BE-PCV14 and lowest performing serotype 3 in Prevenar 13 – Per Protocol Population (N=297)**

| **Anti-PnCPS IgG antibody concentration** | **BE-PCV14 (N=149)**  **N1 (%) [95% CI]** | **Prevenar13 (N=148)**  **N1 (%) [95% CI]** |
| --- | --- | --- |
|  | **Serotype 22F** | **Serotype 6B** |
| **Pre Vaccination (Day 0)** |  |  |
| Seroconverted | 127 (85.2%)  [78.50 : 90.51] | 66 (44.6%)  [36.43 : 52.98] |
| Not Seroconverted | 22 (14.8%)  [9.49 : 21.50] | 104 (70.3%)  [62.21 : 77.50] |
| **Post Vaccination (Day 86)** |  |  |
| Seroconverted | 132 (88.6%)  [82.36 : 93.21] | 108 (73.0%)  [65.06 : 79.94] |
| Not Seroconverted | 17 (11.4%)  [6.79 : 17.64] | 29 (19.6%)  [13.53 : 26.91] |
|  | **Serotype 33F** | **Serotype 6B** |
| **Pre Vaccination (Day 0)** |  |  |
| Seroconverted | 99 (66.4%)  [58.26 : 73.96] | 66 (44.6%)  [36.43 : 52.98] |
| Not Seroconverted | 50 (33.6%)  [26.04 : 41.74] | 104 (70.3%)  [62.21 : 77.50] |
| **Post Vaccination (Day 86)** |  |  |
| Seroconverted | 100 (67.1%)  [58.95 : 74.58] | 108 (73.0%)  [65.06 : 79.94] |
| Not Seroconverted | 49 (32.9%)  [25.42 : 41.05] | 29 (19.6%)  [13.53 : 26.91] |

**Summary of GMC of IgG antibodies against serotypes 22F and 33F in BE-PCV14 and lowest performing serotype 3 in Prevenar 13 – Per Protocol Population (N=297)**

| **IgG antibodies against vaccine serotype** | **BE-PCV14 (N=149)** | **Prevenar13 (N=148)** |
| --- | --- | --- |
|  | **Serotype 22F** | **Serotype 3** |
| **Pre-Vaccination (Day 0)** |  |  |
| N_1_ | 149 | 148 |
| GMC | 0.85 | 0.20 |
| 95 % CI of GMC | (0.75:0.96) | (0.17:0.22) |
| Median | 0.72 | 0.17 |
| (Q1:Q3) | (0.43:1.70) | (0.10:0.42) |
| Range (Min.: Max.) | (0.09:12.89) | (0.02:1.60) |
| **Post-Vaccination (Day 86)** |  |  |
| N_1_ | 149 | 148 |
| GMC | 3.18 | 0.58 |
| 95 % CI of GMC | (2.66:3.80) | (0.52:0.63) |
| Median | 3.85 | 0.60 |
| (Q1:Q3) | (1.73:8.43) | (0.41:0.86) |
| Range (Min.: Max.) | (0.12:23.34) | (0.05:2.66) |
|  | **Serotype 33F** | **Serotype 3** |
| **Pre-Vaccination (Day 0)** |  |  |
| N_1_ | 149 | 148 |
| GMC | 0.59 | 0.20 |
| 95 % CI of GMC | (0.50:0.69) | (0.17:0.22) |
| Median | 0.53 | 0.17 |
| (Q1:Q3) | (0.26:1.25) | (0.10:0.42) |
| Range (Min.:Max.) | (0.03:21.32) | (0.02:1.60) |
| **Post-Vaccination (Day 86)** |  |  |
| N_1_ | 149 | 148 |
| GMC | 1.17 | 0.58 |
| 95 % CI of GMC | (0.91:1.51) | (0.52:0.63) |
| Median | 1.47 | 0.60 |
| (Q1:Q3) | (0.20:5.07) | (0.41:0.86) |
| Range (Min.:Max.) | (0.02:97.34) | (0.05:2.66) |
| **Note:** 95% CI of GMC was calculated by taking the natural log transformation of concentration. 95% CI of GMC ratio was calculated using Wald method on the natural log transformed concentration. GMC = Geometric Mean Concentration; GMCR = Geometric Mean Concentration Ratio. | | |
